# Supplementary figures and images for: Characterization and Genome Analysis of Cladobotryum mycophilum, the Causal Agent of Cobweb Disease of Morchella sextelata in China
Source: J Fungi (Basel). 2023 Mar 27;9(4):411. doi: 10.3390/jof9040411 (PMC10145569; doi:10.3390/jof9040411)

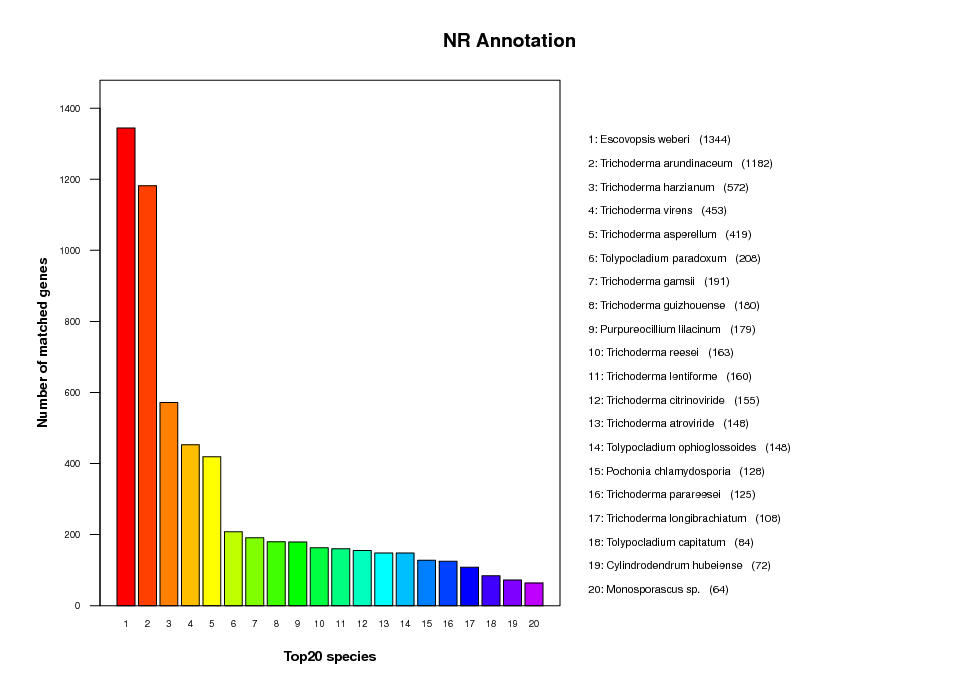

Supplement: Supplementary file 1 [file jof-09-00411-s001.zip › Supplementary Files/Figure S1.png]

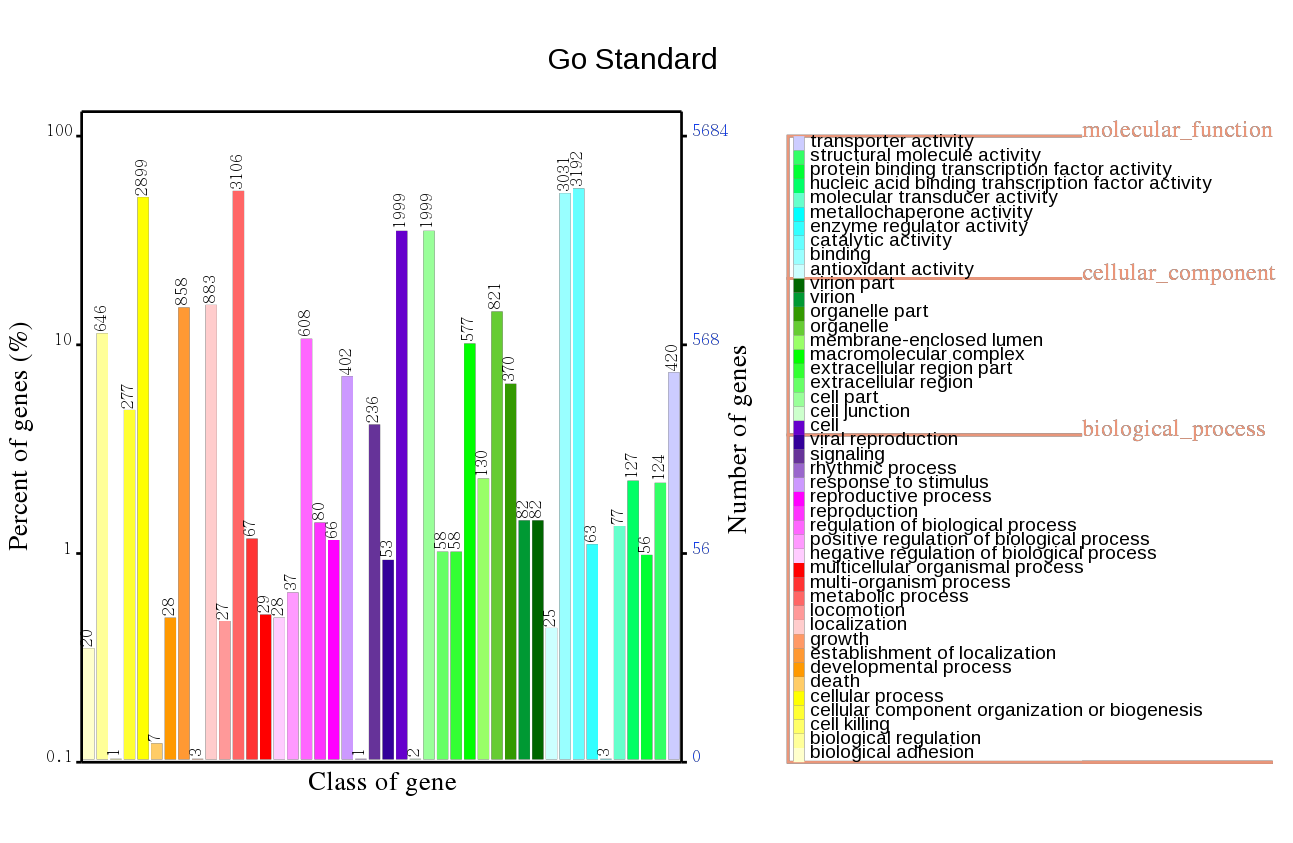

Supplement: Supplementary file 1 [file jof-09-00411-s001.zip › Supplementary Files/Figure S2.png]

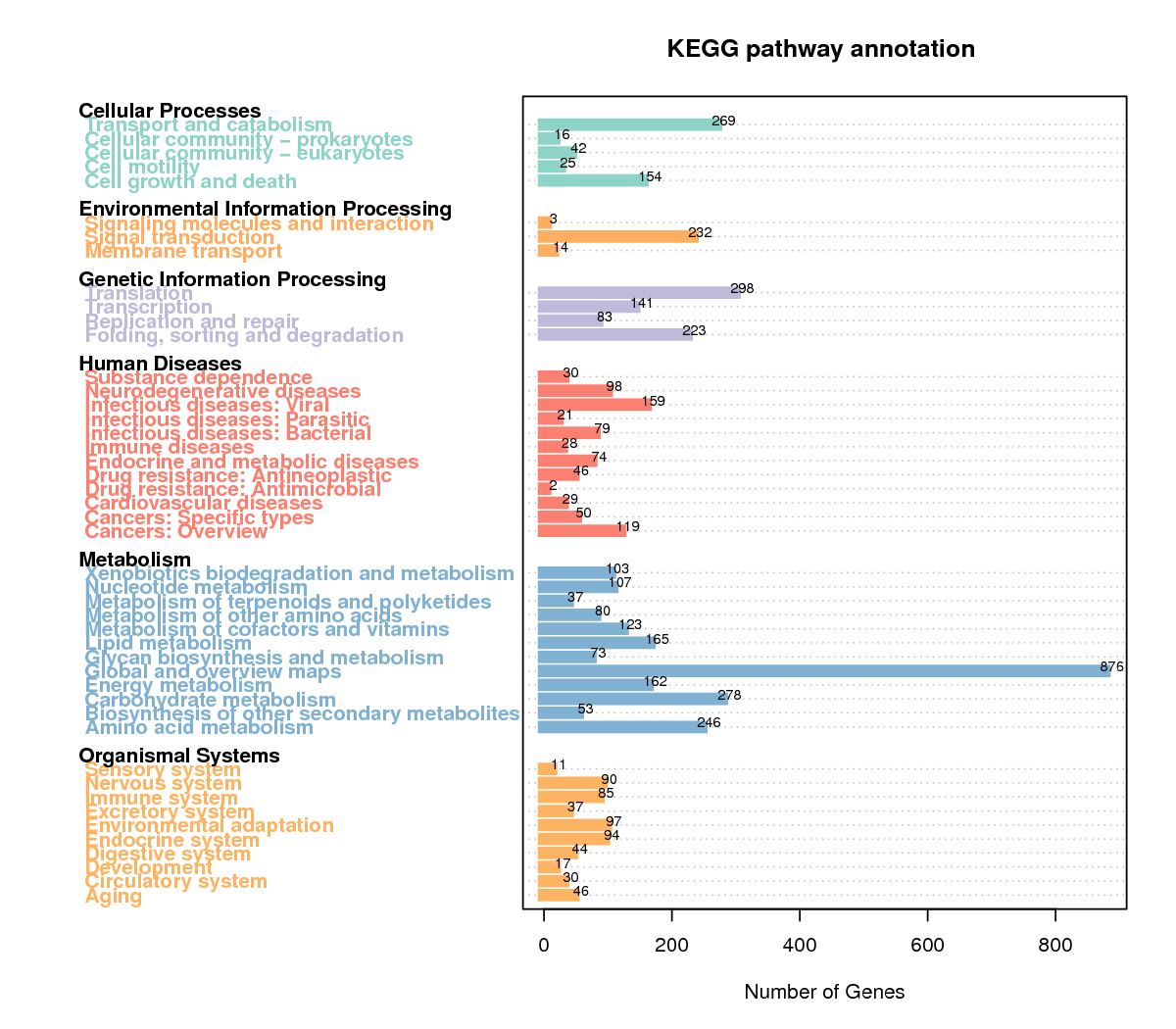

Supplement: Supplementary file 1 [file jof-09-00411-s001.zip › Supplementary Files/Figure S3.png]

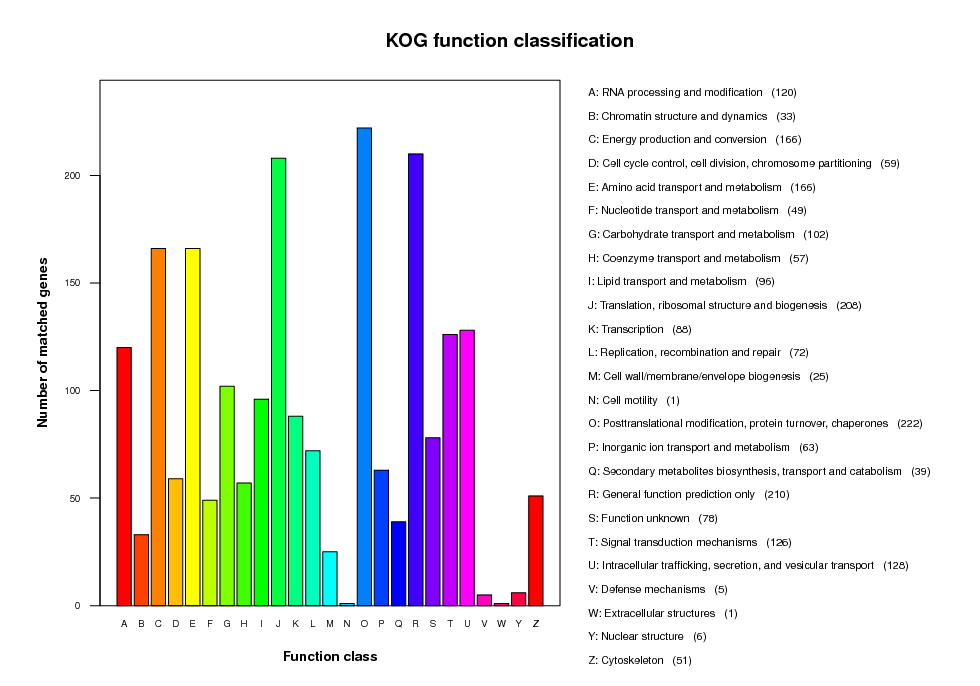

Supplement: Supplementary file 1 [file jof-09-00411-s001.zip › Supplementary Files/Figure S4.png]
